# Hypothalamic volume, sleep, and *APOE* genotype in cognitively healthy adults

**DOI:** 10.1002/alz.70244

**Published:** 2025-05-12

**Authors:** Axel A. S. Laurell, Elijah Mak, Maria‐Eleni Dounavi, Benjamin R. Underwood, Yves Dauvilliers, Robert B. Dudas, Oriane Marguet, Craig W. Ritchie, Ivan Koychev, Brian A. Lawlor, Lorina Naci, Paresh Malhotra, Oriol Grau‐Rivera, Juan Domingo Gispert, John T. O'Brien, for the ALFA study, the PREVENT Dementia Investigators

**Affiliations:** ^1^ Department of Psychiatry University of Cambridge, Addenbrooke's Hospital Cambridge UK; ^2^ Cambridgeshire and Peterborough NHS Foundation Trust, Windsor Research Unit Fulbourn Hospital Cambridge UK; ^3^ Sleep‐Wake Disorders Center Department of Neurology Gui‐de‐Chauliac Hospital, CHU Montpellier Université de Montpellier Montpellier France; ^4^ Department of Psychiatry University of East Anglia Norwich UK; ^5^ Department of Clinical Neurosciences University of Cambridge Cambridge UK; ^6^ School of Medicine University of St Andrews St Andrews UK; ^7^ Department of Psychiatry University of Oxford Warneford Hospital Oxford UK; ^8^ Global Brain Health Institute, Trinity College Dublin Dublin UK; ^9^ Trinity College Institute of Neuroscience, Trinity College Dublin Dublin UK; ^10^ Department of Brain Sciences Imperial College London, Burlington Danes, The Hammersmith Hospital London UK; ^11^ Barcelonaβeta Brain Research Center, Pasqual Maragall Foundation Barcelona Spain; ^12^ Universitat Pompeu Fabra (UPF) Barcelona Spain; ^13^ Hospital del Mar Medical Research Institute Barcelona Spain; ^14^ Servei de Neurologia Hospital del Mar Barcelona Spain; ^15^ Centro de Investigación Biomédica en Red de Fragilidad y Envejecimiento Saludable (CIBER‐FES), Instituto de Salud Carlos III Madrid Spain; ^16^ Centro de Investigación Biomédica en Red de Bioingeniería, Biomateriales y Nanomedicina (CIBER‐BBN) Madrid Spain

**Keywords:** Alzheimer's disease, apolipoprotein E, hypothalamus, neuroimaging, sleep

## Abstract

**INTRODUCTION:**

Sleep dysfunction in those at higher risk of dementia may be associated with early structural changes to the hypothalamus.

**METHODS:**

We used multivariate regression to analyze self‐reported sleep (Pittsburgh Sleep Quality Index [PSQI]) from cognitively healthy participants in the PREVENT Dementia and Alzheimer's and Families (ALFA) studies (*n* = 1939), stratified by apolipoprotein E (*APOE*) genotype as homozygotes, heterozygotes, and non‐carriers. FreeSurfer was used to extract hypothalamic subunit volumes from T1‐weighted magnetic resonance images.

**RESULTS:**

*APOE* ε4 homozygotes had a larger anterior–superior hypothalamus compared to heterozygotes and non‐carriers, an effect which was driven by younger people in the cohort. *APOE* ε4 carriers had a higher PSQI global score after age 55, and smaller anterior–superior and tubular–superior subunits were associated with more sleep disturbances. Sleep duration and efficiency worsened with age, but only in participants with a small anterior–inferior hypothalamus.

**DISCUSSION:**

This suggests that aging and *APOE* ε4 are associated with hypothalamic changes, highlighting mechanisms linking sleep dysfunction to dementia.

**Highlights:**

- Apolipoprotein E (*APOE)* ε4 homozygotes ha a larger anterior–superior hypothalamus.
- *APOE* ε4 carriers have worse sleep, but only after age 55.
- Worse sleep in *APOE* ε4 carriers was associated with smaller hypothalamic subunits.
- Higher age was associated with worse sleep in people with a small hypothalamus.

## 1 BACKGROUND

Disruptions in sleep patterns and sleep–wake circadian rhythms during middle age are associated with an increased risk of displaying abnormal Alzheimer's disease (AD) biomarker results^1^, ^2^, ^3^ and developing AD dementia.^4^ Sleep dysfunction is also a common symptom of established dementia and is associated with significant morbidity.^5^ The etiology of sleep dysfunction in dementia is complex and multifactorial, and remains poorly understood. While neurodegeneration in brain regions responsible for sleep homeostasis may disrupt sleep, studies suggest that sleep dysfunction may, in turn, accelerate amyloid beta (Aβ) accumulation, thereby promoting a neurodegenerative cascade leading to dementia.^2^, ^4^ The hypothalamus is a brain region that contains several different nuclei important for sleep regulation and circadian rhythm.^6^ Although people with mild–moderate AD have a smaller hypothalamus compared to cognitively healthy controls,^7^ very few in vivo studies have investigated how early in the disease process hypothalamic changes occur or how they relate to sleep dysfunction.^8^ How hypothalamic volume is affected by aging and whether changes in the hypothalamus are present in cognitively healthy people at risk of developing dementia are not known.

Apolipoprotein E (*APOE*) ε4 is the most common risk gene for sporadic AD. While *APOE* ε4 heterozygotes have a two to four times increased lifetime risk of AD, homozygotes are at 14 times increased risk, such that 23% to 30% of *APOE* ε4 heterozygotes have developed AD by age 85 compared to 51% to 60% of *APOE* ε4 homozygotes.^9^ Furthermore, *APOE* ε4 homozygotes have detectable changes in cerebrospinal fluid (CSF) Aβ and amyloid positron emission tomography (PET) centiloid scores already in their late 40s with virtually everyone having abnormal results by age 65.^10^ It is less clear whether *APOE* ε4 is also separately associated with common sleep disorders. While some early polysomnography studies found that *APOE* ε4 carriers had a higher rate of obstructive sleep apnea,^11^, ^12^ several subsequent studies and meta‐analyses have since failed to find this effect.^13^, ^14^ *APOE* ε4 also does not increase the risk of narcolepsy^15^ or rapid eye movement (REM) sleep behavior disorder.^16^, ^17^ Despite this, studies using polysomnography have been conflicting, reporting that *APOE* ε4 carriers have lower sleep duration and REM sleep,^18^ lower sleep efficiency,^19^ as well as higher sleep efficiency^20^ compared to non‐carriers. Most cohorts of *APOE* ε4 carriers have included people aged > 60, at which point many *APOE* ε4 carriers have already started accumulating AD pathology. This makes it challenging to differentiate whether sleep dysfunction promotes AD pathology and subsequent cognitive impairment, or whether it is a consequence of early neurodegenerative changes in regions involved in sleep–wake regulation.

Studying the natural history of *APOE* ε4 homozygotes is only possible in large cohort studies considering that only 2% of the UK population are *APOE* ε4 homozygotes, compared to 25% for *APOE* ε4 heterozygotes.^21^ Furthermore, the early onset and slow progression of AD pathology requires a long follow‐up period, or alternatively a wide range of ages, to detect any meaningful changes. In this study, we combine data from two large cohort studies of cognitively healthy participants enriched with *APOE* ε4 carriers, to study the relationships among *APOE* ε4, sleep, and the structure of hypothalamic subunits across an age range of 38 years.

## 2 METHODS

### 2.1 Participants

The full protocol for the PREVENT Dementia^22^, ^23^, ^24^ and ALFA^25^ (for Alzheimer's and Families) cohort studies have been published elsewhere. In PREVENT Dementia, participants aged 40 to 59 were recruited from five different sites in the United Kingdom and Republic of Ireland. Participants were included if they were cognitively healthy, defined as scoring above the age‐dependent cut‐off on the Addenbrooke's Cognitive Examination 3 test.

In the ALFA study, 2743 participants aged 45 to 74 years with increased AD risk based on their AD parental history were recruited from a single site in Spain. Participants were included if they were Spanish‐ and/or Catalan‐speaking, agreed to the study procedures and tests, and had a close relative who could participate in their functional evaluation. Exclusion criteria included scoring below established cut‐offs in cognitive tests (Mini‐Mental State Examination < 26; memory impairment screen < 6; time‐orientation subtest of the Barcelona Test II < 68; semantic fluency (animals) < 12); a Clinical Dementia Rating scale > 0; a major psychiatric, neurological, neurodevelopmental, or physical disorder that could interfere with cognition; and a family history indicative of autosomal dominant AD. The ALFA study consists of a “parent cohort” of enrolled participants (*n* = 2743) and several nested sub‐studies, including the cross‐sectional ALFA‐MRI (*n* ≈ 1600) that included participants who were willing to and had no contraindications to undergoing magnetic resonance imaging (MRI), and the longitudinal ALFA+ (*n* = 420).

Participants in both PREVENT Dementia and ALFA parent cohort underwent genotyping for *APOE* ε4 and were stratified into three groups: non‐carriers (–/–), heterozygotes (ε4/–), and homozygotes (ε4/ε4). Subjects with the genotype ε2/ε4 were excluded from the analysis due to ambiguity in their dementia risk.

**RESEARCH IN CONTEXT**

1. **Systematic review**: A systematic review of Medline and Embase revealed that the relationship between apolipoprotein E (*APOE*) ε4 and changes to sleep is poorly understood. Furthermore, there has been no previous study examining hypothalamic subunit volume in vivo in middle‐aged, cognitively unimpaired, *APOE* ε4 carriers, and very few studies have examined the relationship between hypothalamic structure and sleep.
2. **Interpretation**: Although hypothalamic changes in *APOE* ε4 homozygotes were already detectable in early middle age, the etiology is uncertain. The finding that *APOE* ε4 carriers have sleep changes only in later life suggests that neurodegenerative changes may be causative. Furthermore, our results suggest that the changes to sleep with aging reflect structural changes in the hypothalamus.
3. **Future directions**: Longitudinal studies will enable further description of hypothalamic structural change, and the relationship with sleep and biomarkers of dementia. This may improve our understanding of the pathophysiology of dementia and could generate new treatment targets for sleep disorders.

### 2.2 Self‐reported sleep measurement

Subjective sleep data was assessed using the Pittsburgh Sleep Quality Index (PSQI)^26^ in all participants in the PREVENT Dementia cohort, and in the subset of ALFA‐MRI participants who concurrently participated in the ALFA+ study. The PSQI is a self‐rated questionnaire assessing sleep over the preceding month. It provides a measurement for sleep quality (PSQI component 1), latency (PSQI component 2), duration (PSQI component 3), efficiency (PSQI component 4), disturbances (PSQI component 5), use of sleeping medications (PSQI component 6), and daytime dysfunction (PSQI component 7), with each component being scored on a 4‐point ordinal scale which are then combined to give a global score between 0 and 21. The PSQI was developed and validated as a screening instrument to distinguish between “good” and “poor” sleepers by using the cutoff of > 5 on the PSQI global score.^26^ Scoring above the cutoff on the PSQI global score would usually indicate moderate–severe difficulties (scoring ≥ 2 on a PSQI component) in two to three different areas of sleep. Furthermore, the PSQI global score correlates poorly with objective measures of sleep when treated as a continuous variable.^26^, ^27^ We therefore decided to use the validated binary cutoffs in our analysis, such that a score > 5 on the PSQI global score indicated the presence of sleep dysfunction and a score of ≥ 2 on each PSQI component indicated moderate–severe difficulty in that area of sleep. Depressive symptoms, an important confounder for sleep dysfunction,^28^ was measured using the Center for Epidemiological Studies Depression scale (CES‐D)^29^ in PREVENT Dementia, and by the Hospital Anxiety and Depression Scale (HADS)^30^ in the ALFA‐MRI and ALFA+ sub‐studies. Scores on the scales were harmonized by calculating the percentage of the maximum score for each participant.

### 2.3 Ethical approval

The PREVENT Dementia program received multi‐site ethical approval from the UK London‐Camberwell St Giles National Health Service (NHS) Research Ethics Committee (REC reference: 12/LO/1023, IRAS project ID: 88938), which operates according to the 1975 Declaration of Helsinki (and as revised in 1983). A separate ethical application for Ireland was submitted for the Dublin site, and was reviewed and given a favorable opinion by Trinity College Dublin School of Psychology Research Ethics Committee (SPREC022021‐010) and the St James Hospital/Tallaght University Hospital Joint Research Ethics Committee. All substantial protocol amendments have been reviewed by the same ethics committees and favorable opinion was granted before implementation at sites. The ALFA study was approved by the independent ethics committee “Parc de Salut Mar” (ClinicalTrials.gov identifier NCT01835717).

### 2.4 Image acquisition and analysis

**FIGURE 1:**
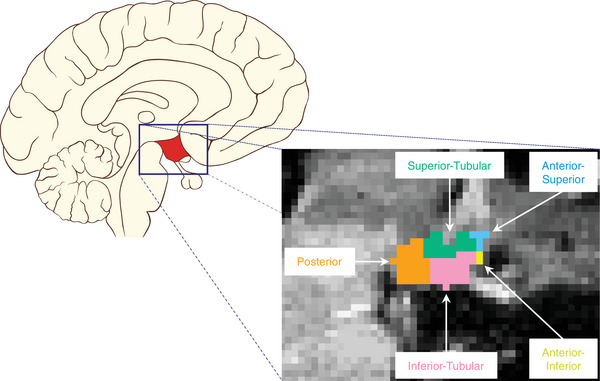
A schematic showing the location of the hypothalamus on a sagittal section of the brain. The hypothalamus was segmented into five subunits using FreeSurfer as shown in the expanded panel.

The PREVENT Dementia protocol included a T1‐weighted magnetization‐prepared rapid gradient echo (MPRAGE) sequence (repetition time = 2.3 seconds, echo time = 2.98 ms, 160 slices, flip angle = 9 degrees, voxel size = 1 mm^3^ isotropic) using the following scanners: 3T Siemens Prisma (Oxford, Edinburgh), Prisma fit (Cambridge), Verio (West London, Edinburgh), and Skyra (Dublin and Edinburgh). The ALFA MRI protocol included a T1‐weighted turbo field echo (TFE) sequence (repetition time = 9.9 ms, echo time = 4.6 ms, flip angle = 8 degrees, voxel size = 0.75 mm^3^ isotropic), which was acquired in a 3T Philips scanner (Ingenia CX, Philips). The volumes of five hypothalamic subunits (anterior–superior, anterior–inferior, superior–tubular, inferior–tubular, posterior), as well as the total intracranial volume (TIV) and total gray matter volume were extracted using an automatic segmentation pipeline in FreeSurfer (version 7.2.0, Figure 1).^31^ This recently developed tool is based on a 3D convolutional neural network, which has been trained and validated against manually labelled T1‐weighted MRI scans.

All images were manually checked for quality control, done blind to knowledge of *APOE* genotype and PSQI scores, and images in which the segmentation algorithm had failed were excluded. Borderline cases were discussed with the developers of the segmentation algorithm. The hypothalamic data were harmonized using combat harmonization to account for the use of multiple MRI scanners, controlling for age, sex, and the presence of *APOE* ε4.^32^ The volume of the right and left hypothalamic subunits were then added for the analysis.

### 2.5 Statistical analysis

The data were analyzed in R Studio (version 2021.09.0) using a series of multivariate regression models, in which predictors were added using a stepwise approach. Robust linear regression models were used to assess the effect of age, sex, TIV, and *APOE* genotype on the volume of each hypothalamic subunit:

Question 1: Does age and sex predict hypothalamic volume?

$$\mathrm{rlm}\left(\left[\mathrm{Hypothalamic}\;\mathrm{subunit}\;\mathrm{volume}\right]\;∼\;\mathrm{TIV}+\mathrm{Age}+\mathrm{Sex}\right)$$

Question 2: Does the interaction between age and sex predict hypothalamic volume?

$$\mathrm{rlm}\left(\left[\mathrm{Hypothalamic}\;\mathrm{subunit}\;\mathrm{volume}\right]\;∼\;\mathrm{TIV}+\mathrm{Age}\;x\;\mathrm{Sex}\right)$$

Question 3: Does *APOE* genotype predict hypothalamic volume?

$$\mathrm{rlm}\left(\left[\mathrm{Hypothalamic}\;\mathrm{subunit}\;\mathrm{volume}\right]\;∼\;\mathrm{TIV}+\mathrm{Age}+\mathrm{Sex}+\mathrm{APOE}\right)$$

Question 4: Does the interaction between *APOE* genotype and age predict hypothalamic volume?

$$\mathrm{rlm}\left(\left[\mathrm{Hypothalamic}\;\mathrm{subunit}\;\mathrm{volume}\right]\;∼\;\mathrm{TIV}+\;\mathrm{Sex}+\mathrm{Age}x\;\mathrm{APOE}\right)$$

The models were applied separately with each of the five hypothalamic subunit volumes as the outcome variable. The output from the five models were then corrected for false discovery rate (FDR) using the Benjamini–Hochberg method.

A series of logistic regression models were used to assess the effect of age, sex, depression, *APOE* genotype, and hypothalamic subunit volume on PSQI global score and PSQI component score:

Question 5: Does age, sex, and depression predict sleep dysfunction?

$$\mathrm{glm}\left(\left[\mathrm{PSQI}\;\mathrm{score}\right]\;∼\;\mathrm{Age}+\mathrm{Sex}+\mathrm{Depression}\right)$$

Question 6: Does *APOE* genotype predict sleep dysfunction?

$$\mathrm{glm}\left(\left[\mathrm{PSQI}\;\mathrm{score}\right]\;∼\;\mathrm{Age}+\;\mathrm{Sex}+\mathrm{Depression}+APOE\right)$$

Question 7: Does the interaction between *APOE* genotype and age predict sleep dysfunction?

$$\mathrm{glm}\left(\left[\mathrm{PSQI}\;\mathrm{score}\right]\;∼\;\mathrm{Sex}+\mathrm{Depression}+\mathrm{Age}\;x\;APOE\right)$$

Question 8: Does hypothalamic subunit volume predict sleep dysfunction?

$$\begin{array}{ccc} & & \mathrm{glm}\left(\left[\mathrm{PSQI}\;\mathrm{score}\right]\;∼\mathrm{Age}+\mathrm{Sex}+\mathrm{Depression}\right.\\ & & \;\left.+\;\left[\mathrm{Hypothalamic}\;\mathrm{subunit}\;\mathrm{volume}\right]\right) \end{array}$$

Question 9: Does the interaction between age and hypothalamic subunit volume predict sleep dysfunction?

$$\begin{array}{ccc} & & \mathrm{glm}\left(\left[\mathrm{PSQI}\;\mathrm{score}\right]\;∼\;\mathrm{Sex}+\mathrm{Depression}+\mathrm{Age}\right.\\ & & \;\left.\times \;\left[\mathrm{Hypothalamic}\;\mathrm{subunit}\;\mathrm{volume}\right]\right) \end{array}$$

Question 10: Does the interaction between *APOE* genotype and hypothalamic subunit volume predict sleep dysfunction?

$$\begin{array}{ccc} & & \mathrm{glm}\left(\left[\mathrm{PSQI}\;\mathrm{score}\right]\;∼\;\mathrm{Age}+\;\mathrm{Sex}+\mathrm{Depression}\right.\\ & & \;\left.+\left[\mathrm{Hypothalamic}\;\mathrm{subunit}\;\mathrm{volume}\right]\;x\;APOE\right) \end{array}$$

The models were first applied with the PSQI global score as the outcome variable. The models were then applied separately with each of the seven PSQI components as the outcome variable. The output from the seven models was then corrected for FDR.

## 3 RESULTS

### 3.1 Demographics

3443 participants were enrolled (PREVENT Dementia: 700, ALFA [parent cohort]: 2743), of which 2169 participants completed MRI scanning (PREVENT Dementia: 666, ALFA‐MRI [sub‐study]: 1503). Hypothalamic segmentation was completed for 2079 (PREVENT Dementia: 601, ALFA‐MRI: 1478), of which 1995 passed manual quality control (PREVENT Dementia: 595, ALFA‐MRI: 1400). *APOE* genotype was available for 1939 participants (PREVENT Dementia: 573, ALFA‐MRI: 1366). There was no difference between the groups in the distribution of age or sex when grouped by *APOE* genotype (Table 1). As expected, the ALFA cohort was significantly older than the PREVENT Dementia cohort (mean: 60 vs. 51 years, *P* < 0.001). The age range of included participants was 40 to 77 years.

**TABLE 1:** There was no significant differences in age, sex, PSQI global score, or depressive symptoms between *APOE* genotype in the cohort with complete volumetric data, or the smaller cohort with complete volumetric and sleep data.

	*APOE* genotype			
	**–/–**	4/–	4/4	
**Volumetric cohort**				
Total *n*	1219	623	97	
PREVENT *n*	363	178	32	
ALFA *n*	856	445	65	
Age (mean [SD])	57.1 (7.30)	57.1 (7.44)	55.9 (6.37)	0.20
Women (*n* [%])	762 (62.5%)	378 (60.7%)	63 (64.9%)	0.62
**Sleep cohort**				
Total *n*	524	283	50	
PREVENT *n*	355	175	29	
ALFA *n*	169	108	21	
Age (mean [SD])	54.5 (6.80)	54.3 (7.13)	53.2 (5.34)	0.29
Women (*n* [(%)]	333 (63.5%)	165 (58.3%)	31 (62.0%)	0.34
PSQI global score, (*n* scoring > 5 [%])	229 (43.7%)	140 (49.5%)	23 (46.0%)	0.29
Depressive symptoms (mean [SD])	13.0 (12.9)	13.7 (13.2)	11.6 (12.6)	0.27

*Note*: Kruskal–Wallis test was used for age and depressive symptoms, while chi‐squared test was used for sex and PSQI global score.

Abbreviations: ALFA, Alzheimer's and Families study; *APOE*, apolipoprotein E; PREVENT, PREVENT Dementia study; PSQI, Pittsburgh Sleep Quality Index; SD, standard deviation.

### 3.2 Effect of age and sex on the hypothalamus

Using multivariate robust linear regression with age, sex, and TIV as predictors revealed that there was a significant association between older age and lower volume of all subunits, except for the anterior–inferior subunit (Table S1 in supporting information). The relationship was particularly strong for the posterior and superior–tubular subunits. All subunits were significantly larger in men than women, even controlling for the effect of TIV, particularly the superior–tubular, inferior–tubular, and posterior subunits. Furthermore, the interaction term (age x sex) was significant for all subunits. This indicates that there was a stronger association in men between older age and lower volume of all hypothalamic subunits, except the anterior–inferior subunit for which the interaction was driven by a small association in women (Figure S1 in supporting information).

### 3.3 Effect of *APOE* ε4 on the hypothalamus

**FIGURE 2:**
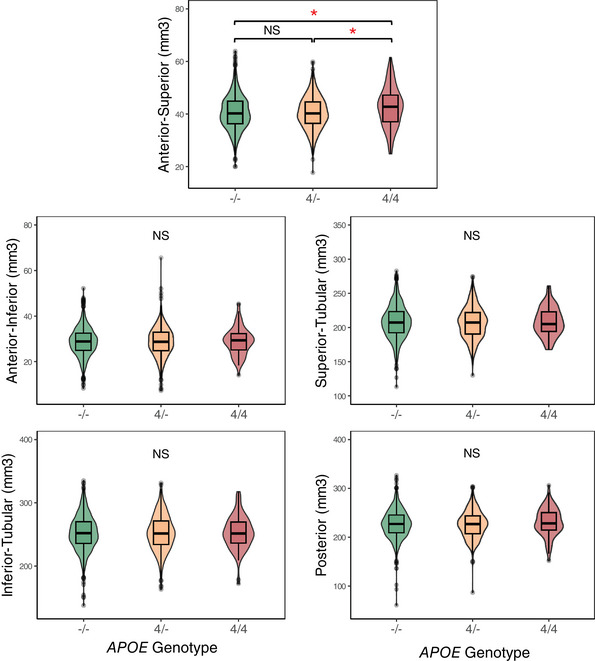
*APOE* ε4 homozygotes (4/4) have a larger anterior–superior hypothalamus compared to heterozygotes (4/–) and non‐carriers (–/–). *P < 0.05, *APOE*, apolipoprotein E; NS, not significant.

**FIGURE 3:**
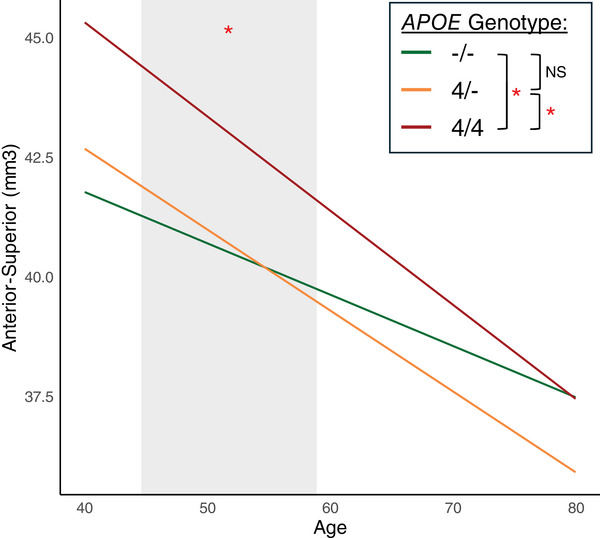
The volume of the anterior–superior hypothalamus was significantly larger in *APOE* ε4 homozygotes (4/4) compared to non‐carriers (–/–) between the ages 45 and 59 (represented by the shaded area). The interaction term (age x *APOE* genotype) was not significant, indicating that the slopes were not significantly different between the different genotypes. However, simple slope analysis revealed that the slopes were significant for non‐carriers (*B* = −0.10, *t* = −3.98, *P* < 0.01), heterozygotes (*B* = −0.17, *t* = −5.33, *P* < 0.01) with a trend for homozygotes (*B* = −0.19, *t* = −1.47, *P* = 0.14). This suggests that homozygotes and heterozygotes may have a greater rate of volume loss with older age in the anterior–superior hypothalamus compared to non‐carriers. *APOE*, apolipoprotein E.

Adding *APOE* genotype as a predictor revealed that *APOE* ε4 homozygotes had a significantly larger anterior–superior hypothalamus compared to heterozygotes (*B* = −2.23[standard error (SE) = 0.68], *P*[FDR] = 0.02) and non‐carriers (*B* = −2.07[SE = 0.66], *P*[FDR] = 0.02; Table 2 and Figure 2). There was also a trend toward a larger superior–tubular, posterior, total hypothalamus, and total gray matter volume in *APOE* ε4 homozygotes. Anterior–superior volume remained significantly larger in *APOE* ε4 homozygotes when adding total gray matter volume as a predictor to the model, indicating that the difference was more pronounced in the anterior–superior hypothalamus than in other gray matter areas of the brain. Furthermore, the volume of the anterior–superior hypothalamus was significantly larger in *APOE* ε4 homozygotes compared to non‐carriers between the ages 45 and 59 (Figure 3), and in homozygotes compared to heterozygotes between the ages 49 and 61, suggesting that the group differences were driven by younger people in the cohort. The interaction term (age x *APOE* genotype) was not significant, indicating that the slopes were not significantly different between the different genotypes. However, simple slope analysis revealed that the within‐group slopes were significant for non‐carriers (*B* = −0.10, *t* = −3.98, *P* < 0.01) and heterozygotes (*B* = −0.17, *t* = −5.33, *P* < 0.01) with a trend for homozygotes (*B* = −0.19, *t* = −1.47, *P* = 0.14). This suggests that homozygotes and heterozygotes may have a greater rate of volume loss with older age in the anterior–superior hypothalamus compared to non‐carriers.

**TABLE 2:** The volume of the hypothalamic subunits, stratified by *APOE* genotype.

	Mean volume (mm^3^ [SD])		
	–/–	4/–	4/4
**Anterior**–**superior**	41 (6)	40 (6)	43 (8)^*^, ^‡^
**Anterior**–**inferior**	29 (6)	29 (7)	29 (6)
**Superior–tubular**	208 (24)	207 (23)	209 (21)
**Inferior–tubular**	253 (27)	253 (28)	253 (26)
**Posterior**	227 (28)	226 (27)	231 (28)
**Whole hypothalamus**	758 (74)	755 (70)	765 (68)
**Total gray matter volume**	613344 (58381)	612153 (56216)	620239 (54561)
**Total intracranial volume**	1453409 (164661)	1457573 (171391)	1469949 (161525)

*Note*: Group differences were analyzed using robust linear regression, controlling for age, sex, total intracranial volume, and false discovery rate.

Abbreviations: *APOE*, apolipoprotein E; FDR, false discovery rate; SD, standard deviation.

* *P*(FDR) < 0.05 ε4/ε4 versus –/–.

^‡^ *P*(FDR) < 0.05 for ε4/ε4 versus ε4/–.

### 3.4 Effect of age, sex, and *APOE* genotype on PSQI scores

Complete data on PSQI scores, depression, and *APOE* genotype was available for 963 participants (PREVENT Dementia: 658, ALFA+ [sub‐study]: 305). Of these, 592 were non‐carriers, 318 *APOE* ε4 heterozygotes, and 53 *APOE* ε4 homozygotes. Logistic regression models with age, sex, and depression as the predictors revealed an association between more depressive symptoms and higher PSQI global score and all PSQI component scores, indicting more sleep dysfunction (all *P*[FDR] < 0.001). Older age was associated with a lower frequency of sleep disturbances (*B* = 0.03[SE = 0.01], *P*[FDR] = 0.04) and a higher use of sleeping medications (*B* = −0.09[SE = 0.02], *P*[FDR] < 0.001). Furthermore, women had a significantly longer sleep latency (*B* = 0.55[SE = 0.18], *P*[FDR] = 0.01) and higher use of sleeping medications (*B* = 0.79[SE = 0.27], *P*[FDR] = 0.01) than men, indicating more sleep dysfunction (Figure S2 in supporting information). When adding *APOE* genotype as a predictor to the model, this was not significantly associated with changes to the PSQI global or component scores. There was also no significant interaction effect between age and *APOE* genotype.

### 3.5 Effect of hypothalamic volume on PSQI scores

**FIGURE 4:**
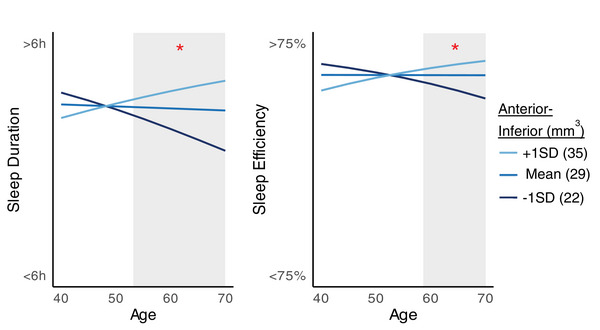
The volume of the anterior–inferior hypothalamic subunit significantly moderated the relationship between age and sleep duration (*B* = 0.005[SE = 0.002], *P*[FDR] = 0.02) and sleep efficiency (*B* = 0.006[SE = 0.002], *P*[FDR] = 0.04). The shaded regions indicate the age at which the interaction was significant. A smaller anterior–inferior volume was significantly associated with shorter sleep duration after 53 years, and with lower sleep efficiency after 59 years. FDR, false discovery rate; SD, standard deviation; SE, standard error.

Complete data on PSQI scores, depression, *APOE* genotype, and hypothalamic volume was available for 857 participants (PREVENT Dementia: 559, ALFA+ [sub‐study]: 298). Of these, 524 were non‐carriers, 283 *APOE* ε4 heterozygotes, and 50 *APOE* ε4 homozygotes. Logistic regression models with age, sex, depression, and hypothalamic subunit volume as predictors revealed that a larger anterior–inferior subunit was associated with a shorter sleep latency (*B* = 0.04[SE = 0.01], *P*[FDR] = 0.02) and longer sleep duration (*B* = 0.03[SE = 0.01], *P*[FDR] = 0.03; Figure S3 in supporting information). Furthermore, the volume of the anterior–inferior hypothalamic subunit significantly moderated (significant age x volume interaction term) the relationship between age and sleep duration (*B* = 0.005[SE = 0.002], *P*[FDR] = 0.02) and sleep efficiency (*B* = 0.006[SE = 0.002], *P*[FDR] = 0.04; Figure 4). There was also a trend for the volume of the superior–tubular hypothalamus to moderate the relationship between age and sleep latency (*B* = 0.001[SE = 0.0005], *P*[FDR] = 0.06). Johnson–Neyman intervals showed that a smaller anterior–inferior volume was significantly associated with shorter sleep duration after 53 years, and with lower sleep efficiency after 59 years. This indicates increasing age is associated with more sleep dysfunction, but only in people with a smaller hypothalamus, indicating that the sleep dysfunction may be driven by hypothalamic volume loss. There was no significant interaction effect between hypothalamic volume and *APOE* genotype.

### 3.6 Subgroup analysis

### 3.7 Age

**FIGURE 5:**
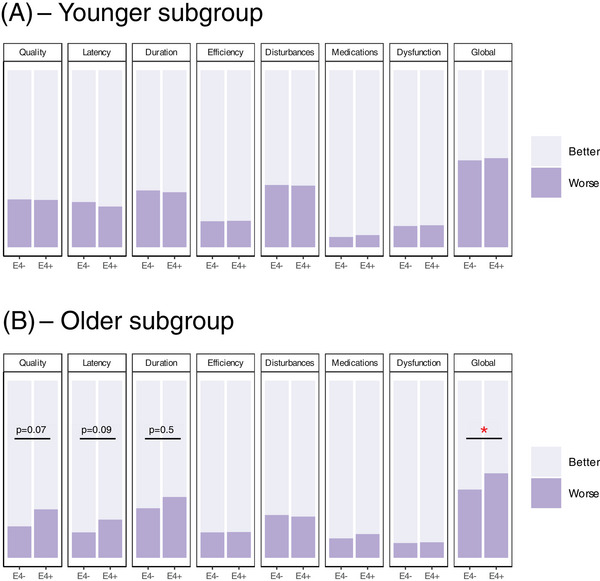
A, There was no difference in sleep between *APOE* ε4 carriers (ε4/– and ε4/ε4 combined) and non‐carriers in the younger subgroup. B, *APOE* ε4 carriers had a higher PSQI global score (*B* = 0.43[SE = 0.22], *P* = 0.047) compared to non‐carriers in the older subgroup, which was driven by worse sleep quality (*B* = −0.66[SE = 0.26], *P*[FD]) = 0.07) and sleep latency (*B* = −0.58[SE = 0.26], *P*[FDR]) = 0.09). *P < 0.05. *APOE*, apolipoprotein E; FDR, false discovery rate; PSQI, Pittsburgh Sleep Quality Index; SE, standard error.

**FIGURE 6:**
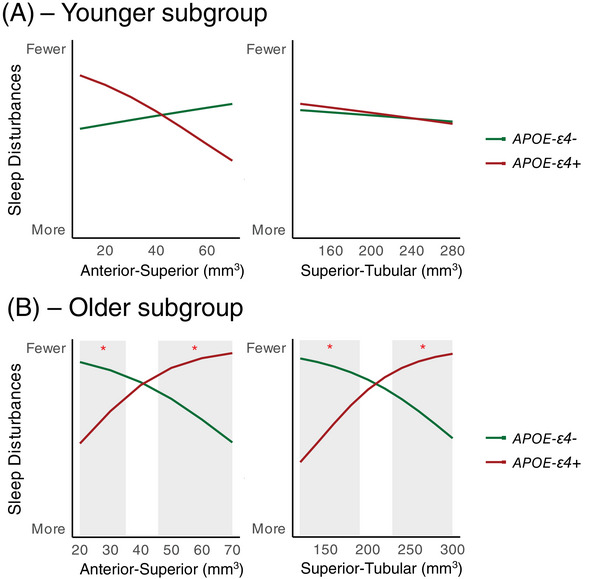
A, There was no significant interaction between *APOE* genotype and hypothalamic subunit volume for the PSQI global or component scores in the younger subgroup. B, In the older subgroup, a smaller anterior–superior and superior–tubular hypothalamus was associated with more sleep disturbances (PSQI component 5) in *APOE* ε4 carriers, while larger subunits were associated with more sleep disturbances in non‐carriers. The shaded regions indicate the volume at which the interaction term (volume x *APOE* genotype) was significant. *APOE*, apolipoprotein E; PSQI, Pittsburgh Sleep Quality Index.

Because age is the most important predictor of AD pathology, we undertook a subgroup analysis to assess sleep in younger versus older participants. The cohort with complete PSQI scores, depression, *APOE* genotype, and hypothalamic volume was split into two groups based on the median age and analyzed separately (“older”: ≥ 55 years, *n* = 437; “younger”: < 55 years, *n* = 420). Due to the small number of *APOE* ε4 homozygotes in each group (older: *n* = 19, younger: *n* = 31), we combined *APOE* ε4 homozygotes and heterozygotes for this analysis. In both subgroups, depressive symptoms were associated with more sleep dysfunction (*P*[FDR] < 0.01 for PSQI global and all component scores). Within the younger subgroup, age, sex, or *APOE* ε4 status did not significantly predict PSQI global or component scores (Figure 5A). However, in the older subgroup, increasing age was associated with less sleep disturbances (*B* = 0.19[SE = 0.04], *P*[FDR] < 0.001), and more frequent use of sleeping medications (*B* = −0.12[SE = 0.04], *P*[FDR] < 0.01). In addition, *APOE* ε4 carriers had a higher PSQI global score (*B* = 0.43[SE = 0.22], *P* = 0.047) compared to non‐carriers in the older subgroup, which was driven by worse sleep quality (*B* = −0.66[SE = 0.26], *P*[FDR] = 0.07) and sleep latency (*B* = −0.58[SE = 0.26], *P*[FDR] = 0.09; Figure 5B). This effect remained significant when adding the volume of each hypothalamic subunit to the model. In the older subgroup, *APOE* genotype significantly moderated the association between the anterior–superior (*B* = 0.13[SE = 0.04], *P*[FDR] = 0.02) and superior–tubular (*B* = 0.04[SE = 0.01], *P*[FDR] = 0.006) volume and the frequency of sleep disturbances (significant volume x *APOE* interaction term; Figure 6B). Johnson–Neyman intervals showed that this interaction was significant at both larger and smaller anterior–superior and superior–tubular subunits. This indicates that smaller hypothalamic subunits were associated with more sleep disturbances in *APOE* ε4 carriers, while larger subunits were associated with more sleep disturbances in non‐carriers. There was no significant interaction in the younger subgroup (Figure 6A).

### 3.8 Sex

Because we observed significant differences between men and women in relation to both hypothalamic volume and PSQI component scores, we conducted a subgroup analysis based on sex. In men, older age was associated with shorter sleep latency (*B* = 0.06[SE = 0.02], *P*[FDR] = 0.05) and anterior–superior volume also moderated (significant age x volume interaction term) the association between age and PSQI global score (*B* = −0.006[SE = 0.003], *P* = 0.03). In women, higher age was associated with fewer sleep disturbances (*B* = 0.05[SE = 0.02], *P*[FDR] = 0.008) and increasing sleep medication use (*B* = −0.08[SE = 0.02], *P*[FDR] = 0.002). There were no significant age x volume interactions in women.

### 3.9 Body mass index

Because higher body mass index (BMI) is associated with an increase in hypothalamic volume,^33^ we investigated whether this influenced our results. BMI was available in a subgroup of our sample (*n* = 572). In this subgroup, there was a trend for *APOE* ε4 heterozygotes to have a larger anterior–superior hypothalamus than heterozygotes (*B* = −2.74[SE = 1.27], *P* = 0.08) and non‐carriers (*B* = −2.60[SE = 1.21], *P* = 0.08), controlling for age, sex, and TIV. Adding BMI as a predictor to the model did not change the *P* value of this association. However, there was a trend toward higher BMI being associated with larger anterior–inferior (*B* = 0.10[SE = 0.05], *P*[FDR] = 0.09) and posterior (*B* = 0.39[SE = 0.19], *P*[FDR] = 0.09) subunits.

## 4 DISCUSSION

In this study we combined cross‐sectional data from two large cohort studies with an age range of 38 years, to investigate whether *APOE* genotype and older age were associated with sleep dysfunction and structural differences in the hypothalamus. We found that *APOE* ε4 homozygotes had a significantly larger anterior–superior hypothalamus, an effect driven by differences in younger participants. Furthermore, there was a trend for the association between older age and lower anterior–superior volume to be stronger for *APOE* ε4 homozygotes and heterozygotes than non‐carriers, suggesting that this subunit may be vulnerable to volume loss from AD pathology. Several hypothalamic nuclei are important for regulating sleep including the ventrolateral preoptic nucleus within the anterior–superior subunit, the suprachiasmatic nucleus within the anterior–inferior subunit, and the dorsomedial nucleus and lateral hypothalamus within the superior–tubular subunit.^34^ Consistent with this, we found that a smaller anterior–superior hypothalamus is associated with a higher frequency of sleep disturbances in older *APOE* ε4 carriers (homozygotes and heterozygotes combined). We also found that older age was associated with shorter sleep duration and lower sleep efficiency in the full cohort, but only in people with a smaller anterior–inferior hypothalamus. Overall, these results suggest that sleep dysfunction with aging, and in *APOE* ε4 carriers, may be driven by hypothalamic volume loss.

### 4.1 *APOE* ε4 and hypothalamic volume

The etiology of the larger anterior–superior volume in *APOE* ε4 homozygotes is not clear but may be due to neuroinflammation, developmental changes, or other unknown factors affecting the hypothalamus. *APOE* ε4 homozygotes have abnormal levels of AD biomarkers in the CSF from their late 40s,^10^ and the *APOE* ε4 allele is associated with a proinflammatory phenotype.^35^ Cognitively healthy participants with positive AD biomarkers have increased levels of inflammatory cytokines in the CSF, indicating that neuroinflammation may be an early feature of AD, which may possibly contribute to an increase in size of affected brain regions.^36^ We also observed that larger anterior–superior and superior–tubular hypothalamic subunits were associated with a higher frequency of sleep disturbances in older *APOE* ε4 non‐carriers, providing further support that hypothalamic hypertrophy may represent early pathological changes. In the PREVENT Dementia cohort, we have previously shown that *APOE* ε4 carriers have increased cerebral perfusion,^37^, ^38^, ^39^ a smaller molecular layer of the hippocampus,^40^ increased cerebral microbleeds,^41^ and that a larger posterior thalamus is associated with worse immediate recall.^42^ Within the combined PREVENT Dementia and ALFA cohorts, *APOE* ε4 carriers had a steeper decline in white matter integrity from age 60,^43^ although no volumetric differences were found using voxel‐based morphometry.^44^ The decline in white matter integrity is consistent with our finding that *APOE* ε4 homozygotes and heterozygotes have a trend toward increased rate of volume loss in the anterior–superior hypothalamus with increasing age. Other studies have found that the volume of the hypothalamus is reduced in people with mild–moderate dementia due to AD compared to controls,^7^ with the anterior–superior hypothalamus being the most affected subunit.^31^ *Post mortem* studies have also demonstrated that AD pathology can be found throughout the hypothalamus.^45^, ^46^ Although there are no similar studies in *APOE* ε4 carriers, the volume of the hypothalamus is affected in presymptomatic mutation carriers for amyotrophic lateral sclerosis,^47^ and in prodromal Huntington's disease, up to 15 years before predicted clinical diagnosis.^48^

The increased volume of the anterior–superior hypothalamus may also be consistent with the antagonistic pleiotropy hypothesis, which proposes that prevalent risk alleles such as *APOE* ε4 are associated with an evolutionary advantage early in life while being harmful later in life.^49^ Consistent with this theory, *APOE* ε4 carriers perform better on cognitive tasks,^50^, ^51^, ^52^ have a lower risk of proximal colorectal cancer,^53^ and increased survival from melanoma.^35^ Furthermore, *APOE* ε4 carriers with low amounts of AD neuropathology have an overall reduced mortality risk compared to non‐carriers.^54^ A possible explanation could be that the immunomodulatory effect of *APOE* ε4 contributes to neuroinflammation and an increased risk of AD,^55^, ^56^ but may also infer a protective effect in other circumstances such as cancer.^35^ However, it is unclear how a larger anterior–superior hypothalamus would contribute to an evolutionary advantage, and in vivo studies are lacking.

Other factors may also influence hypothalamic volume. For example, a higher BMI is associated with an increase in volume and mean diffusivity of the hypothalamus,^33^, ^57^, ^58^ with the tubular–superior and posterior subunits particularly affected.^33^ BMI was only available for a subgroup of our cohort (*n* = 572), and although we observed a positive trend between BMI and the volumes of the anterior–inferior and posterior subunits, including BMI as a covariate did not affect our finding of a larger anterior–superior hypothalamus in *APOE* ε4 homozygotes. Again, the etiology of increased hypothalamic volume with a higher BMI is not clear, although hypothalamic inflammation has been associated with obesity in rodents.^59^, ^60^

### 4.2 Sleep and dementia risk

Although sleep dysfunction is associated with an increased risk of developing dementia,^4^ the underlying mechanism is poorly understood, and it is unclear whether it represents an early biomarker of neurodegeneration in the brain areas responsible for sleep homeostasis. Furthermore, sleep dysfunction itself may accelerate the neurodegenerative process. In AD, sleep dysfunction is associated with reduced glucose uptake in the hypothalamus,^8^ and increased CSF levels of the hypothalamic peptides melanin‐concentrating hormone^61^ and orexin‐A.^8^, ^62^, ^63^, ^64^, ^65^, ^66^ Several meta‐analyses have found that *APOE* ε4 carriers do not have an increased risk of obstructive sleep apnea,^13^, ^14^ and polysomnography studies have found that *APOE* ε4 carriers have both a lower^19^ and higher^20^ sleep efficiency compared to non‐carriers. However, neither of these studies corrected for depressive symptoms, which is an important confounding factor for sleep dysfunction.^28^ We found that *APOE* ε4 was associated with more sleep dysfunction, but only in participants over 55 years. Furthermore, smaller anterior–superior and tubular–superior subunits were associated with a higher frequency of sleep disturbances in *APOE* ε4 carriers, indicating that hypothalamic changes may partially explain the differences in sleep between *APOE* ε4 carriers and non‐carriers.

We also present the first in vivo evidence that a lower volume of the anterior–inferior hypothalamus is associated with shorter sleep duration and longer sleep latency. The anterior–inferior subunit contains the suprachiasmatic nucleus, and lesions to this structure in monkeys cause complete disruption to the sleep–wake cycle, underlining its importance for circadian rhythm.^67^ Furthermore, increasing age was associated with shorter sleep duration and lower sleep efficiency, but only in the people with a small anterior–inferior hypothalamus. At younger ages, the volume of the anterior–inferior hypothalamus had no relationship with sleep, indicating that acquired, rather than inherent, structural changes in this subunit may be contributing to sleep dysfunction. This relationship becomes significant at 52.3 years, which is roughly consistent with the estimates of when AD pathology is thought to start accumulating in the brain.^68^

We also observed a significant effect of sex on hypothalamic volume, with men having significantly larger hypothalamic volume compared to women, but also show a greater age‐associated volume loss for almost all subunits. This is consistent with imaging^7^, ^69^ and pathology studies^70^, ^71^ showing that the hypothalamus is more affected by AD pathology in men than women, despite men having a larger hypothalamus than women in midlife. Whether this has any sex‐specific impact on symptoms, function, or outcomes in AD requires further study.

### 4.3 Limitations

Due to the cross‐sectional nature of this study, associations should not be interpreted as causal and longitudinal studies are required to confirm that the age‐associated changes in volume represent brain atrophy. The study is also at risk of selection bias as the lifetime risk of AD rises to 28% for *APOE* ε4 homozygotes and 7% for *APOE* ε4 heterozygotes by age 75.^9^ Because all participants in our cohort studies were cognitively intact, the factors which protected them from developing AD may also have influenced sleep and hypothalamic volume. Finally, we measured self‐reported sleep using the PSQI, which may correlate poorly with objective measures of sleep in older adults,^26^, ^27^ although the observed effects of age, sex, and depression on sleep is consistent with results from polysomnography studies.^72^

### 4.4 Conclusion

To the best of our knowledge, this is the first study to use in vivo structural MRI to study the relationship between the structure of the hypothalamic subunits and sleep dysfunction in *APOE* ε4 carriers. Using cross‐sectional data from a large cohort of cognitively healthy participants across 38 years, we demonstrate that *APOE* ε4 homozygotes have an increased volume of the anterior–superior hypothalamus in mid‐life, which decreases more quickly than non‐carriers with age. In addition, we show that older *APOE* ε4 carriers have more sleep dysfunction than non‐carriers, and that smaller anterior–superior and tubular–superior subunits in this group are associated with more sleep disturbances. In the full cohort, older age was associated with shorter sleep duration and lower sleep efficiency, but only in people with a small anterior–inferior hypothalamus. Overall, these results indicate that age‐related changes to sleep may be driven by neurodegenerative changes in the hypothalamus. Longitudinal studies will need to confirm how these findings relate to biomarkers for AD, neuroinflammation, and the sleep dysfunction which is prominent in people with established dementia.

## CONFLICT OF INTEREST STATEMENT

A.A.S.L.’s post is funded by a grant from Altos labs and is a member of the National Institute for Health and Care Research (NIHR) Dementia Portfolio Development Group. Data from this project will also be included as part of a PhD thesis by A.A.S.L. B.R.U. is the R&D director at his trust, CRN lead for dementia for the East of England, and national CRN lead for stratified medicine in dementia. He is the vice‐chair of the faculty of old age psychiatry in the Royal College of Psychiatry. His post is part funded by a generous donation from Gnodde Goldman Sachs gives. He has served in a paid role on advisory boards for Lilly and TauRx. J.O.B. has acted as a consultant for TauRx, Novo Nordisk, Biogen, Roche, Lilly, GE Healthcare, and Okwin and received grant or academic in kind support from Avid/ Lilly, Merck, UCB, and Alliance Medical. M.E.D. is supported by an Alzheimer's Society Fellowship (grant number 612). I.K. declares funding for this work through Medical Research Council Dementias Platform UK grant (MR/T033371/1), National Institute Health Research (personal award NIHR301616 and Oxford Health Biomedical Research Centre). I.K. also declares paid consultancies for Johnson and Johnson and speaker fees from Novo Nordisk. He has received grant funding from Novo Nordisk. He is a paid medical advisor for digital technology (Five Lives SAS, Cognes, Paloma Health, Lola Cares) and biotechnology (CFDX Ltd) companies in the dementia field. J.D.G. is currently a full‐time employee at AstraZeneca. E.M., Y.D., R.B.D., O.M., C.W.R., B.A.L., L.N., P.M., O.G.R. have nothing to disclose. Author disclosures are available in the supporting information.

## CONSENT STATEMENT

All human subjects included in this study provided informed consent.

## Supporting information



Supporting Information

Supporting Information

## Data Availability

The PREVENT dataset is available to access through a data request on the study website (www.preventdementia.co.uk); on the Alzheimer's Disease Data Initiative (ADDI) platform baseline dataset DOI: https://doi.org/10.34688/PREVENTMAIN_BASELINE_700V1; Dementia Platforms UK (DPUK); and the Global Alzheimer's Association Network (GAAIN).
