# Supplementary material for: Hypothalamic volume, sleep, and APOE genotype in cognitively healthy adults
Source: Alzheimers Dement. 2025 May 12;21(5):e70244. doi: 10.1002/alz.70244 (PMC12069023; doi:10.1002/alz.70244)
Supplement: Supplementary file 1 — Supporting Information [file ALZ-21-e70244-s001.docx]

| **Hypothalamic subunit** | **B (Age)** | **Standard Error** | **t** | **F** | **p** |
| --- | --- | --- | --- | --- | --- |
| Anterior-Superior | -0.134 | 0.020 | -6.877 | 47.169 | **<0.001** |
| Anterior-Inferior | 0.027 | 0.019 | 1.467 | 2.153 | 0.143 |
| Superior-Tubular | -0.482 | 0.063 | -7.602 | 57.519 | **<0.001** |
| Inferior-Tubular | -0.166 | 0.071 | -2.344 | 5.468 | **0.024** |
| Posterior | -0.668 | 0.073 | -9.133 | 82.317 | **<0.001** |

**Table S1.** Increased age was associated with a smaller volume of all hypothalamic subunits, except for the anterior-inferior subunit. The robust linear regression models were adjusted for sex, total intracranial volume, and p-values were corrected false discovery rate across the five subunits.


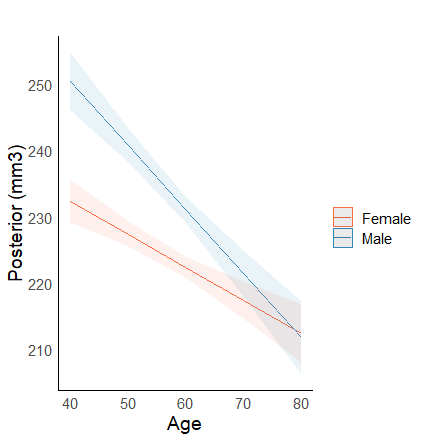

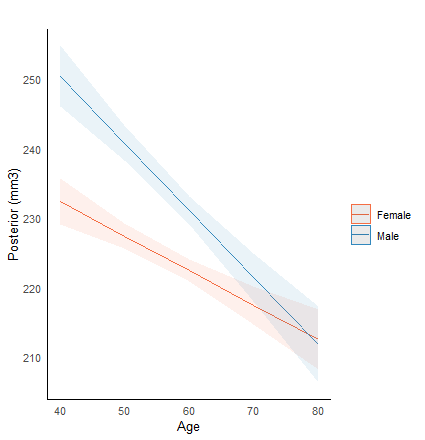

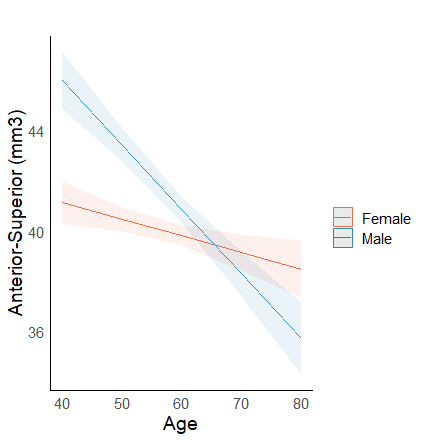

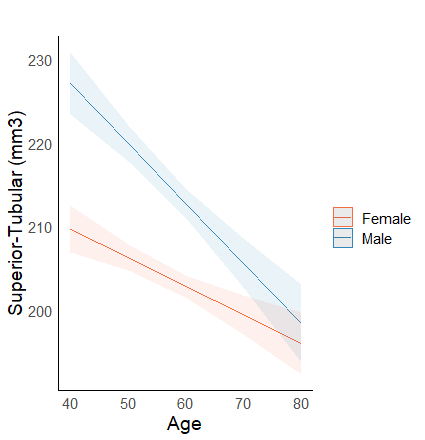

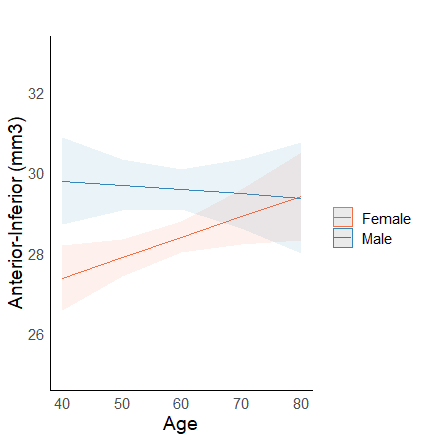

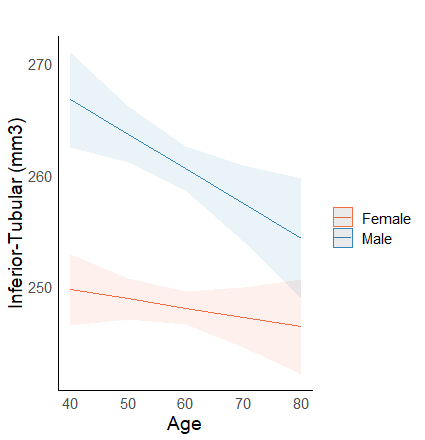


**

**

*

NS

**

*

*

NS

Women

Men

**

**

**Figure S1.** There was a significant interaction between age and sex for all hypothalamic subunits (All p<0.001). There was a relationship between higher age and lower volume for all subunits in both men and women, except for the anterior-inferior subunit which had a positive relationship with age in women and no relationship in men, and the inferior-tubular subunit where there was a relationship between higher age and lower volume in men but not women. Slopes: **p<0.01, *p<0.05, NS = not significant.


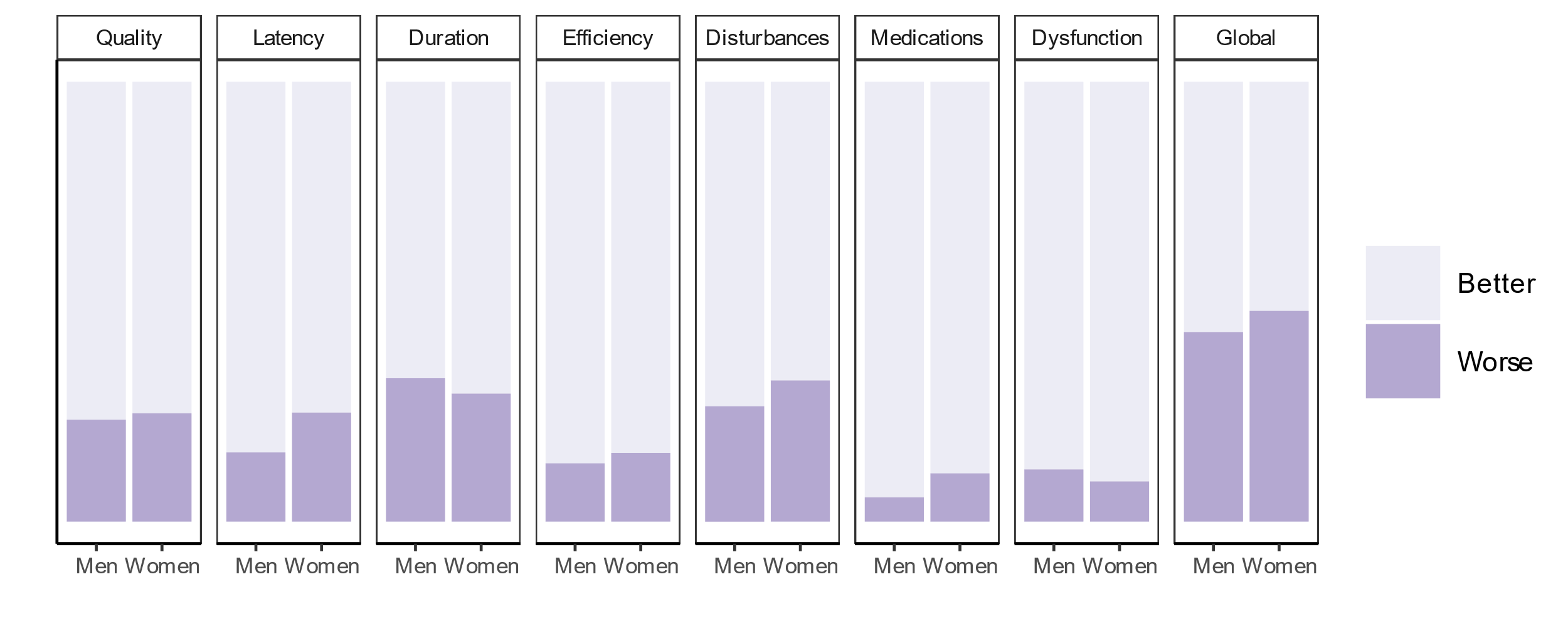


*

*

**Figure S2.** Women had a significantly longer sleep latency (B=0.55(SE=0.18), p(FDR)=0.01) and higher use of sleeping medications (B=0.79(SE=0.27), p(FDR)=0.01) than men, indicating more sleep dysfunction. *p<0.05.

**Figure S3.** Increased volume of the anterior-inferior hypothalamus was associated shorter sleep latency (B=0.04(SE=0.01), p(FDR)=0.02) and longer sleep duration (B=0.03(SE=0.01), p(FDR)=0.03) using logistic regression, controlling for age, sex and depression. *p<0.05.

Anterior-Inferior (mm^3^)

Sleep Duration

Sleep Latency

*


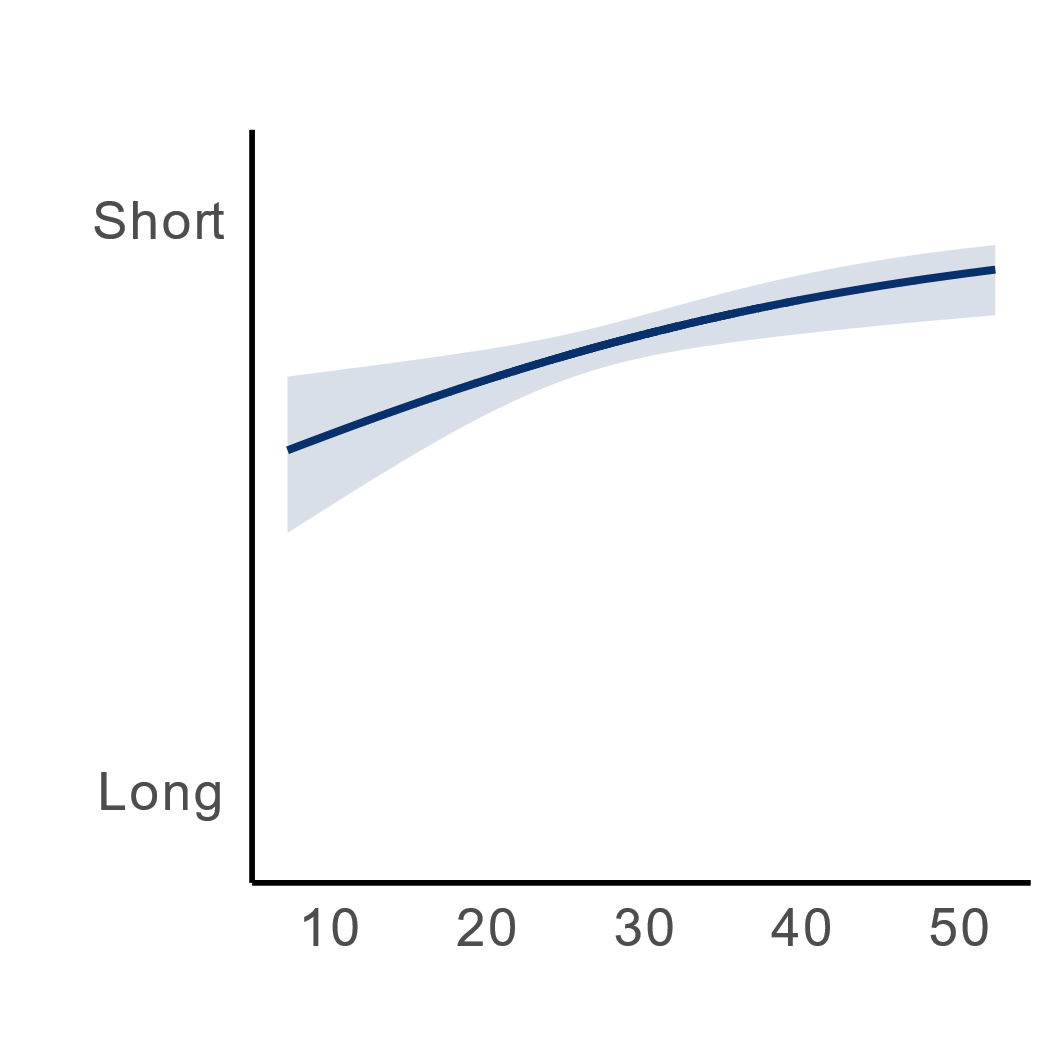

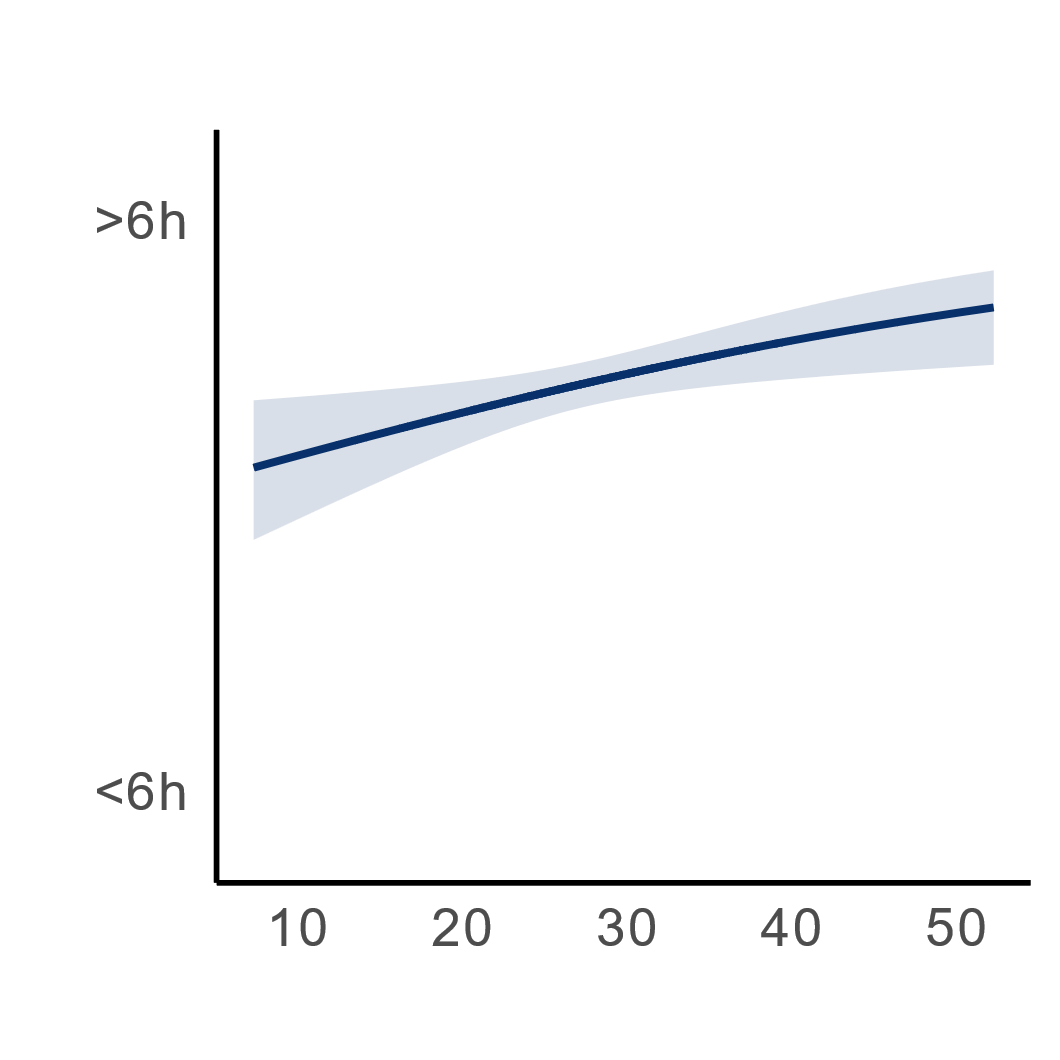


*
